# Investigation of the relationships between perceived causes of COVID-19, attitudes towards vaccine and level of trust in information sources from the perspective of Infodemic: the case of Turkey

**DOI:** 10.1186/s12889-021-11262-1

**Published:** 2021-06-23

**Authors:** Şemsi Nur Karabela, Filiz Coşkun, Haydar Hoşgör

**Affiliations:** 1 Vocational School of Health Services, University of Health Sciences Turkey, Mekteb-i Tibbiye-Şahane Campus 34668 Uskudar, Istanbul, Turkey; 2 Institute of Graduate Studies, Istanbul University-Cerrahpaşa, Istanbul, Turkey; 3 Vocational School of Health Services, Usak University, Usak, Turkey

**Keywords:** Pandemic, COVID-19, COVID-19 vaccine, Infodemic

## Abstract

**Background:**

The main purpose in this study carried out from the perspective of infodemic was to investigate the relationships between individuals’ perceived causes of COVID-19, their attitudes towards vaccine and their levels of trust in information sources in terms of various descriptive characteristics.

**Methods:**

In this cross-sectional and correlational study conducted with 1216 individuals from different provinces of Turkey, the Perception of Causes of COVID-19 (PCa-COVID-19) Scale was used. In addition, a questionnaire including the participants’ descriptive characteristics, their attitudes towards vaccine and their level of trust in information sources about Covid-19 was used.

**Results:**

The mean age of the participants was 35.9 ± 12.3 years. Of them, 62.5% were women, 59.0% were married, and 62.1% were university graduates. As for their view of having the Covid-19 vaccine, 54.1% thought to have it, 16.2% did not think, and 29.7% were undecided. Although the correlation was not significant, of the participants, those who considered having vaccination mostly trusted YouTube as their source of information. Of the participants, those whose level of trust in government institutions and health professionals was high displayed significantly more favorable attitudes towards vaccine. The participants obtained the highest mean score from the Conspiracy Theories subscale of the PCa-COVID-19 scale. There was a positive and low-level relationship between attitudes towards COVID-19 vaccine, and the Conspiracy Theories (r: 0.214) and Faith Factors (r: 0.066) sub-dimensions of the PCa-COVID-19 Scale.

**Conclusions:**

The level of vaccine hesitancy in Turkey is at an alarming level, and the virus is defined by moderate conspiracy theories. In this context, in the fight against infodemic, it is critical to implement mechanisms that can reveal misinformation and to plan initiatives that can increase the health literacy levels of societies.

**Supplementary Information:**

The online version contains supplementary material available at 10.1186/s12889-021-11262-1.

## Background

Due to the terrifying increase in COVID-19 cases and COVID-19-related deaths, the World Health Organization declared that this viral disease became a pandemic on March 11, 2020 [1]. According to the report released by Johns Hopkins Coronavirus Resource Center in May 2021, Turkey ranks fifth after the United States, India, Brazil and France with its more than five million confirmed cases [2].

The rapid spread of the COVID-19 disease around the world causes new data about the pandemic to emerge and spread constantly. Although people try to change their life styles according to these new data and to adapt to them, this process cannot always proceed in a healthy way due to the bombardment of false and misleading information they are exposed to [3].

The concept of infodemic [4] derived from the words “information” and “pandemic” in English, took its place again on the agenda at the global level after Dr. Tedros Adhanom Ghebreyesus, the director-general of the World Health Organization (WHO), stated that “We are fighting not only a pandemic, but also an infodemic” in February 2020 [5]. This concept is defined by the World Health Organization as “a massive collection of information emerging during an epidemic, some of which is true, some of which is wrong, that spreads rapidly like a virus and complicates the health organization” [6].

From the historical perspective, deceptive and fake news is not a new phenomenon. However, the most important difference between today and the past is the propagation speed of information and the existence of many different platforms that facilitate this propagation. Traditional and digital media environments, which positively affect citizen participation, have a very important place in democracy; on the other hand, they may spread infodemic rapidly [7].

Infodemic, which refers to the rapid spread of fake news or false information to the whole world through both social media platforms and traditional mass media such as television, radio and newspapers, causes masses to display inappropriate behaviors, which jeopardizes the efforts of governments and health officials to manage COVID-19, and can cause panic and xenophobia [8]. Infodemic also harms the physical and mental health of societies, increases stigmatization, decreases the intention to have vaccination, and speeds up the spread of the pandemic [9].

COVID-19, which deeply affects all areas of life, has become a determinant of individuals’ and societies’ behaviors and thought patterns with the effect of infodemic. In many countries such as Canada, Germany, Poland, the United Kingdom and the United States, the number of and violence of protests against mandatory use of masks and closures have increased. In some countries, there has been fake news that there are no surgical masks or drugs such as hydroxychloroquine. Many patients in Europe have refused to take ibuprofen due to the misbelief that it worsens the symptoms of COVID-19 [10].

It is known that in addition to fake news, some conspiracy theories have led to the rapid spread of infodemic. According to the findings of a study carried out in the United States, 60% of individuals were of the opinion that COVID-19 was produced in a laboratory, and the risks related to the virus were exaggerated [11]. In another study conducted to investigate conspiracy theories on COVID-19, it was reported that the related theories were as follows: it is the exaggeration of governments / media, it is China’s biological weapon, it is the strategy of controlling the population, and it is the plague of the modern age due to sins committed by people [12]. In the literature, it is reported that misinformation such as “the COVID-19 virus dies at 27°C”, “certain foods strengthen the immune system and prevent the disease”, and “the virus affects mostly older people rather than infants and children” diffuses like a drop of ink in water [13, 14].

In the management of the COVID-19 pandemic, it is very important that the sources used by people to learn about the COVID-19 pandemic should be reliable, because it can be argued that a possible environmental insecurity may further increase the inclination towards anti-vaccination in societies. For example, in a study conducted during the Ebola epidemic, tweets containing misleading medical information reached approximately 15 million potential readers in just 1 week [15]. Therefore, in order to minimize the infodemic, which is considered as dangerous as this virus in today’s digital age, the information provided to the masses must be accurate, up-to-date, complete, always accessible and based on scientific resources [16, 17]. In a study conducted in Nigeria during the pandemic, the most widely used sources of information regarding COVID-19 disease were traditional media, social media, internet, Nigeria Center for Disease Control, family / friends and political leaders respectively [12]. In a study conducted with 907 people in Turkey, the sources of information most trusted by individuals regarding COVID-19 were university / training-research hospitals in the city where they are located, the World Health Organization, and the Coronavirus Scientific Committee of the Ministry of Health of the Republic of Turkey respectively [18].

This study was aimed at investigating the relationships between individuals’ perceived causes of COVID-19, their attitudes towards vaccine and their levels of trust in information sources from the perspective of infodemic.

## Methods

### Participants and design

The population of this cross-sectional and correlational study consists of individuals over the age of 18 living in Turkey. For individuals to participate in the study, they were required to be literate and to have a smartphone enabling them to access the internet and WhatsApp application. Due to the large population of the study, the cluster sampling method was used in this study [19]. Considering that there are seven geographical regions in Turkey, a city with a metropolitan municipality status was chosen from each region to represent that region. In this context, Istanbul from the Marmara Region, Ankara from the Central Anatolia Region, İzmir from the Aegean Region, Adana from the Mediterranean Region, Şanlıurfa from the Southeast Anatolia Region, Samsun from the Black Sea Region, and Van from the Eastern Anatolia Region were selected. An e-survey link was shared with individuals living in these cities via Google Forms (https://forms.gle/zxcjasDJW87DdAVAA). While the data were collected, at least one contact person was selected from each city. In this study, carried out between February 01, 2021 and February 28, 2021, 18 survey forms were excluded because there had missing answers and therefore 1216 forms were evaluated within the scope of the study. The permission to conduct the study was obtained from the ethics committee of the University of Health Sciences on January 22, 2021. After all the participants were told that the data collected would only be used for scientific purposes, their informed consent was obtained. This study was carried out in accordance with the 1964 Helsinki Declaration and the ethical standards of the National Research Committee.

### Instruments

The tools used to collect the study data were the “Perception of Causes of COVID-19” Scale, and a questionnaire questioning the participants’ descriptive characteristics, their attitudes towards vaccine and their level of trust in information sources about COVID-19 were used.

#### Perception of causes of COVID-19 (PCa-COVID-19) scale

The scale was adapted by Geniş et al. [20] from a scale developed by Çırakoğlu for swine influenza (H1N1) [21]. Geniş et al. also performed the validity and reliability study of the PCa-COVID-19 (Scope Validity Index: 0.84). The scale has 14 items and three sub-dimensions namely “Conspiracy Theories”, “Environmental Factors”, “Faith Factors”. Responses given to the items are rated on a five-point Likert type scale ranging from 1 (strongly disagree) to 5 (strongly agree). The Conspiracy Theories sub-dimension consists of six items questioning conspiracy beliefs (biological warfare, vaccine sales, etc.) frequently expressed in the media as the causes of the disease. The Environmental Factors sub-dimension consists of five items pointing to the social and physical environment (unhealthy diet, global warming, pollution of natural resources, etc.) as possible causes of the COVID-19 outbreak. The Faith Factors sub-dimension, which consists of three statements, is related to the perceptions of religious and divine explanations (that the epidemic is in our destiny, God’s wrath against social deterioration, etc.) as the causes of COVID-19. There are no reverse scored items in the scale. The sum of the scores of the items devided by the number of the items in that sub-dimension yields the overall score of that sub-dimension which ranges between 1 and 5. The higher the score is the higher the level of the perception in that sub-dimension is. The Cronbach’s Alpha internal reliability coefficient of the original scale is 0.88 for the overall scale and 0.96, 0.85 and 0.90 for the Conspiracy Theories, Environmental Factors and Faith Factors sub-dimensions respectively.

#### Descriptive characteristics of the participants

This section consists of nine items questioning the participants’ age, sex, marital status, education level, place of residence, employment status, being diagnosed with COVID-19 or not, and the diagnosis and death of a relative.

#### Participants’ attitudes towards vaccine

This section consists of one question: “Do you intend to get COVID-19 vaccine?” answered as “Yes”, “No”, “Undecided”.

#### Level of trust in COVID-19 information sources

This section includes items questioning the participants’ level of trust in information sources such as social media, YouTube, WhatsApp, websites, newspapers, television, friends / relatives, government institutions and health professionals in terms of their attitudes towards vaccine.

### Statistical analysis

Data analysis was performed using the IBM SPSS (The Statistical Package for the Social Sciences) 22.0. Descriptive statistics such as frequency, percentage, arithmetic mean, standard deviation, minimum and maximum values were used within the scope of the study. While independent samples t-test, one-way analysis of variance (ANOVA) and Tukey’s post-hoc test were used to determine the differences in the analysis of continuous variables, and Pearson r was used to determine the relations between the variables. The prerequisite for using these parametric tests is that the data should be normally distributed (Table 3). Also, the Chi-Square test was used in the analysis of categorical variables. In this context, Kolmogorov-Smirnov test was used to check whether the data were normally distributed, and it was found that the normal distribution value was lower than the statistical significance level (*p* < 0.05). Therefore, Kurtosis-Skewness values ​​were used for the normal distribution. As is known, when the number of participants in the study group is high, the kurtosis-skewness value of ±1.96 provides the assumption of normality [22]. In addition, *p* value was accepted as 0.05 for the significance of the data to be evaluated at 95% confidence interval. Microsoft Office Excel 2016 package program was used for drawing the figures.

## Results

As is seen in Table 1 which includes the descriptive characteristics of the participants, their mean age was 35.9 ± 12.3, and of them, 26.1% were in the 18–25 group, 62.5% were women, 59.0% were married, 62.1% were university graduates. 56.7% lived in Istanbul, 60.6% worked full time, 82.9% were not diagnosed with COVID-19 positive, and more than 70% had relatives diagnosed with COVID-19 positive, It has been determined that approximately 30% of the participants have relatives who died due to Covid-19.

**Table 1:** Descriptive Characteristics of the Participants (*N* = 1216)

Descriptive Characteristics	Groups	n (Number)	(Percentage) %
**Age** (Mean: 35.9 ± 12.3) years	18–25	317	26.1
	26–33	205	16.9
	34–41	281	23.1
	42–49	249	20.5
	≥50	164	13.5
**Sex**	Female	760	62.5
	Male	456	37.5
**Marital status**	Single	498	41.0
	Married	718	59.0
**Educational level**	Primary education	42	3.5
	High school	125	10.3
	Bachelor’s degree	755	62.1
	Postgraduate	294	24.2
**City of residence**	İstanbul	690	56.7
	Ankara	158	13.0
	İzmir	98	8.1
	Adana	46	3.8
	Samsun	71	5.8
	Şanlıurfa	60	4.9
	Van	93	7.6
**Employment status**	Full-time	737	60.6
	Part-time	54	4.4
	Retired	53	4.4
	Student	230	18.9
	Unemployed	142	11.7
**Were you diagnosed with COVID-19**	Yes	208	17.1
	No	1008	82.9
**Any of your relatives diagnosed with COVID-19**	Yes	854	70.2
	No	362	29.8
**Death of any relative in your family from COVID-19?**	Yes	359	29.5
	No	857	70.5

**Fig. 1:**
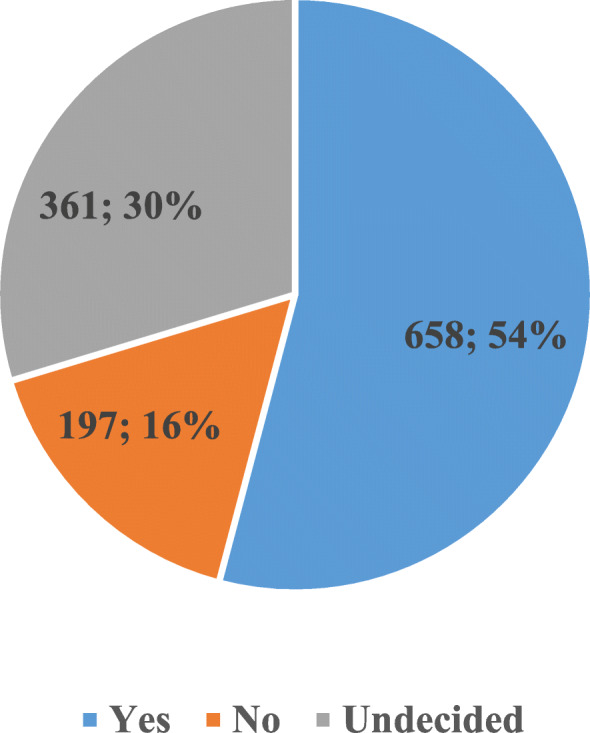
Participants’ Attitudes towards COVID-19 Vaccine

As is seen in Fig. 1 which reflects the participants’ attitudes towards the COVID-19 vaccine, of them, 54% thought of being vaccinated, 16% did not think, and 30% were undecided.

**Fig. 2:**
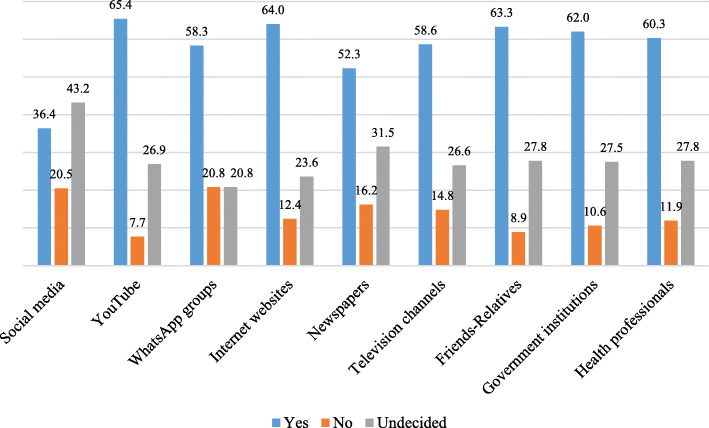
Attitudes Displayed towards Vaccine by the Participants regarding the COVID-19 Information Sources

According to Fig. 2, the source of information trusted most was YouTube for the participants who stated that they would be vaccinated, WhatsApp groups for the participants who stated that they would not be vaccinated and social media for the participants who stated that they were undecided.

According to the findings of the Chi-Square analysis given in Table 2, of the participants, those who stated that they would have the COVID-19 vaccine did not trust social media sites such as Facebook, Twitter and Instagram (p: 0.005), moderately trusted newspapers (p: 0.047) and trusted websites (p: 0.010), televisions (p: 0.000), friends / relatives (p: 0.029), government institutions (p: 0.000) and health professionals (p: 0.000) very much. On the other hand, there was no significant relationship between the level of trust in YouTube (p: 0.361) and WhatsApp groups (p: 0.347), and the participants’ attitudes towards vaccine.

**Table 2:** The Relationship Between the Level of Trust in COVID-19 Information Sources and Attitudes towards Vaccine

Do you trust social media?	Thinking of Getting COVID-19 Vaccine			X^**2**^	***P***
	Yes (%)	No (%)	Undecided (%)		
Very much	36.4	20.5	43.2	14.887	0.005*
Moderately	52.7	15.3	32.1		
Not at all	58.8	17.5	23.7		
**Do you trust YouTube?**	**Yes (%)**	**No (%)**	**Undecided (%)**	4.344	0.361
Very much	65.4	7.7	26.9		
Moderately	54.0	15.0	31.0		
Not at all	53.7	18.1	28.2		
**Do you trust WhatsApp?**	**Yes (%)**	**No (%)**	**Undecided (%)**	4.462	0.347
Very much	58.3	20.8	20.8		
Moderately	53.6	14.7	31.6		
Not at all	54.3	17.4	28.4		
**Do you trust websites?**	**Yes (%)**	**No (%)**	**Undecided (%)**	13.227	0.010*
Very much	64.0	12.4	23.6		
Moderately	52.9	15.2	31.9		
Not at all	55.0	21.6	23.4		
**Do you trust the newspapers?**	**Yes (%)**	**No (%)**	**Undecided (%)**	9.597	0.047*
Very much	52.3	16.2	31.5		
Moderately	56.5	14.2	29.3		
Not at all	48.7	21.3	30.0		
**Do you trust TV?**	**Yes (%)**	**No (%)**	**Undecided (%)**	49.64	0.000*
Very much	58.6	14.8	26.6		
Moderately	56.3	12.1	31.6		
Not at all	42.9	31.2	26.0		
**Do you trust friends / relatives?**	**Yes (%)**	**No (%)**	**Undecided (%)**	10.778	0.029*
Very much	63.3	8.9	27.8		
Moderately	51.1	16.8	32.1		
Not at all	58.4	16.4	25.2		
**Do you trust government institutions?**	**Yes (%)**	**No (%)**	**Undecided (%)**	72.931	0.000*
Very much	62.0	10.6	27.5		
Moderately	50.7	16.2	33.1		
Not at all	36.1	39.1	24.8		
**Do you trust healthcare professionals?**	**Yes (%)**	**No (%)**	**Undecided (%)**	56.229	0.000*
Very much	60.3	11.9	27.8		
Moderately	42.9	23.0	34.1		
Not at all	31.0	40.5	28.6		

As is seen in Table 3 which includes descriptive statistics regarding the causes of COVID-19, the participants had a moderate level of perception of causes of COVID-19 (2.80 ± 0.66). As for the sub-dimensions, the participants’ level of perception was moderate for the “Conspiracy Theories” (2.93 ± 1.01) and “Environmental Factors” (2.88 ± 0.87) sub-dimensions, and low for the “Faith Factors” (2.41 ± 1.11) sub-dimension. The participants chose the “Strongly Agree” option for the following statements: “This disease was produced as a biological weapon - Conspiracy Theories”, “This disease is a consequence of an unhealthy lifestyle - Environmental Factors” and “This epidemic is in our destiny - Faith Factors”.

**Table 3:** Descriptive Statistics For Perception of Causes of COVID-19

Descriptive Statistics	General Scale	Sub-Dimensions		
	Perception of Causes of Covid-19	Conspiracy Theories	Environmental Factors	Faith Factors
Mean*	2.80	2.93	2.88	2.41
**Standard Deviation**	0.66	1.01	0.87	1.11
**Skewness**	−0.02	0.01	0.10	0.39
**Kurtosis**	0.28	−0.47	− 0.43	− 0.76
**Minimum**	1.00	1.00	1.00	1.00
**Maximum**	5.00	5.00	5.00	5.00
**Cronbach’s Alpha**	0.83	0.79	0.84	0.81

*1.00–1.80: Very Low, 1.81–2.60: Low, 2.61–3.40: Moderate, 3.41–4.20: High, 4.21–5.00: Very High

As is seen in Table 4, there is a weak positive correlation between the participants’ attitudes towards COVID-19 vaccine and the Conspiracy Theories and Faith Factors sub-dimensions but no significant relationship between their attitudes and the Environmental Factors sub-dimension. In other words, as the participants’ perceptions of conspiracy theories and faith factors increased, they displayed positive attitudes towards vaccine. While there was no significant correlation between the participants’ ages and the Conspiracy Theories and Faith Factors sub-dimensions, there was a weak negative relationship between the Environmental Factors sub-dimension and their attitudes towards vaccine. In other words, as the participants’ age increased, their levels of perceptions of environmental factor and attitudes towards vaccine decreased.

**Table 4:** The Relationship Between Perception of Causes of COVID-19, Attitudes towards Vaccine and Age Variables

Variables		1	2	3	4
**Conspiracy Theories (1)**	Pearson Correlation				
	*p*-value				
**Environmental Factors (2)**	Pearson Correlation	0.037			
	*p*-value	0.195			
**Faith Factors (3)**	Pearson Correlation	0.290^**^	0.156^**^		
	*p*-value	**0.000**	**0.000**		
**Vaccine Attitude (4)**	Pearson Correlation	0.214^**^	0.013	0.066^*^	
	*p*-value	**0.000**	0.660	**0.022**	
**Age (5)**	Pearson Correlation	−0.024	− 0.182**	0.043	− 0.176**
	*p*-value	0.399	**0.000**	0.133	**0.000**

**. Correlation is significant at 0.01 (Two-tailed)

*. Correlation is significant at 0.05 (Two-tailed)

In Table 5, the results of the t-test on whether there were significant differences between the descriptive characteristics of the participants and the sub-dimensions of the COVID-19 Perception of Causes Scale are given. According to the table, the mean score the female participants obtained from the Environmental Factors sub-dimension (2.93 ± 0.85) was significantly higher than was that obtained by the male participants (*p* < 0.05). The mean score the single participants obtained from the Environmental Factors sub-dimension (3.04 ± 0.86) was significantly higher than was that obtained by the married participants (*p* < 0.05). While the participants who were not diagnosed with COVID-19 positive obtained a significantly higher mean score from the Environmental Factors sub-dimension (2.91 ± 0.88), the participants who were diagnosed with COVID-19 positive obtained a significantly higher mean score from the Faith Factor sub-dimension (2.62 ± 1.17) (*p* < 0.05). On the other hand, the participants who had a relative diagnosed with COVID-19 obtained a significantly higher mean score from the Faith Factor sub-dimension (2.47 ± 1.11) than did the participants who did not have a relative diagnosed with COVID-19 (*p* < 0.05).

**Table 5:** Pair Group Comparison of Descriptive Characteristics of Participants and Sub-Dimensions of the Perception of Causes of COVID-19 Scale

Scale and Sub-scales		Groups	n	M	SD	LL	UL	***P***
**Sex**	Conspiracy Theories	Female	760	3.02	0.95	0.10	0.33	0.937
		Male	456	2.99	1.04	0.10	0.34	
	Environmental Factors	Female	760	2.93	0.85	0.04	0.24	0.008*
		Male	456	2.79	0.90	0.03	0.24	
	Faith Factors	Female	760	2.49	1.12	0.10	0.36	0.238
		Male	456	2.47	1.14	0.11	0.36	
**Marital status**	Conspiracy Theories	Single	498	2.93	1.04	−0.13	0.10	0.840
		Married	718	2.94	0.98	− 0.13	0.10	
	Environmental Factors	Single	498	3.04	0.86	0.18	0.37	0.000*
		Married	718	2.77	0.86	0.18	0.37	
	Faith Factors	Single	498	2.39	1.10	−0.15	0.10	0.734
		Married	718	2.41	1.11	−0.15	0.10	
**Have You Been Diagnosed with COVID-19?**	Conspiracy Theories	Yes	208	3.05	1.04	0.00	0.30	0.058
		No	1008	2.91	1.00	−0.01	0.30	
	Environmental Factors	Yes	208	2.74	0.81	−0.29	−0.03	0.014*
		No	1008	2.91	0.88	−0.29	−0.04	
	Faith Factors	Yes	208	2.62	1.17	0.09	0.42	0.003*
		No	1008	2.36	1.09	0.08	0.43	
**Status of your relatives regarding diagnosis with COVID-19**	Conspiracy Theories	Yes	854	2.93	1.01	−0.14	0.11	0.861
		No	362	2.94	0.99	−0.13	0.11	
	Environmental Factors	Yes	854	2.90	0.86	−0.04	0.17	0.260
		No	362	2.83	0.91	−0.05	0.17	
	Faith Factors	Yes	854	2.47	1.11	0.08	0.35	0.002*
		No	362	2.26	1.09	0.08	0.35	
**Did Any of your Relatives die Due To COVID-19?**	Conspiracy Theories	Yes	359	2.96	1.03	−0.09	0.16	0.611
		No	857	2.92	1.00	−0.09	0.16	
	Environmental Factors	Yes	359	2.94	0.88	−0.01	0.20	0.090
		No	857	2.85	0.87	−0.01	0.20	
	Faith Factors	Yes	359	2.47	1.14	−0.04	0.23	0.171
		No	857	2.38	1.09	−0.04	0.24	

* *p* < 0.05 n, M, SD, LL, and UL represent Number, Mean, Standard Deviation, Lower Limit, and Upper Limit, respectively

In Table 6, the results of ANOVA on whether there is a significant differencies between the descriptive characteristics of the participants and the sub-dimensions of the COVID-19 Perception of Causes of COVID-19 are given. Accordingly, in terms of the place of residence, while the participants living in Van obtained significantly higher mean scores from the Conspiracy Theories subscale of the Perception of Causes of COVID-19 scale (3.14 ± 0.94), the participants living in Şanlıurfa obtained significantly higher mean scores from the Faith Factors subscale of the Perception of Causes of COVID-19 scale (3.06 ± 0.96) (*p* < 0.05). The comparison of the participants in terms of their education levels demonstrated that primary school graduates obtained significantly higher mean scores from the Conspiracy Theories (3.29 ± 1.02) and Faith Factors (3.12 ± 1.13) subscales than did those with a master degree (*p* < 0.01). As for the employment status, the unemployed participants obtained significantly higher mean scores from the Conspiracy Theories (3.17 ± 1.00) and Faith Factors (2.97 ± 1.15) subscales than did the full-time working participants (*p* < 0.05).

**Table 6:** Multi-Group Comparison of the Descriptive Characteristics of the Participants and the Sub-Dimensions of the Perception of Causes of COVID-19 Scale

Scale and Sub-scales		Groups	n	M	SD	LL	UL	***P***	Post-Hoc
**City of residence**	Conspiracy Theories	İstanbul (a)	690	2.92	1.03	2.85	3.00	0.000*	g > c
		Ankara (b)	158	3.02	0.95	2.87	3.17		
		İzmir (c)	98	2.43	0.97	2.23	2.62		
		Adana (d)	46	3.04	0.91	2.77	3.31		
		Samsun (e)	71	3.11	0.91	2.90	3.33		
		Şanlıurfa (f)	60	3.06	0.96	2.81	3.31		
		Van (g)	93	3.14	0.94	2.95	3.33		
	Environmental Factors	İstanbul	690	2.90	0.88	2.84	2.97	0.880	
		Ankara	158	2.86	0.85	2.72	2.99		
		İzmir	98	2.85	0.92	2.66	3.03		
		Adana	93	2.91	0.85	2.74	3.08		
		Samsun	71	2.78	0.84	2.58	2.98		
		Şanlıurfa	60	2.79	0.80	2.58	3.00		
		Van	46	2.86	0.89	2.59	3.12		
	Faith Factors	İstanbul (a)	690	2.41	1.14	2.33	2.50	0.001*	f > c
		Ankara (b)	158	2.48	1.09	2.31	2.65		
		İzmir (c)	98	1.93	0.94	1.74	2.11		
		Adana (d)	93	2.49	1.07	2.27	2.71		
		Samsun (e)	71	2.43	1.02	2.19	2.67		
		Şanlıurfa (f)	60	2.66	1.10	2.38	2.95		
		Van (g)	46	2.52	1.00	2.23	2.82		
**Educational Level**	Conspiracy Theories	Primary education (a)	42	3.29	1.02	2.97	3.61	0.000*	a > d
		High school (b)	125	3.04	0.98	2.87	3.22		
		Bachelor’s degree (c)	755	2.91	1.04	2.82	3.00		
		Postgraduate (d)	294	2,86	0.90	2.73	2.98		
	Environmental Factors	Primary education (a)	42	2.84	0.79	2.81	3.39	0.870	
		High school (b)	125	2.86	0.83	2.70	2.98		
		Bachelor’s degree (c)	755	2.87	0.88	2.80	2.95		
		Postgraduate (d)	294	2.85	0.84	2.75	2.98		
	Faith Factors	Primary education (a)	42	3.12	1.13	2.77	3.47	0.000*	a > d
		High school (b)	125	2.75	1.16	2.55	2.96		
		Bachelor’s degree (c)	755	2.22	1.06	2.13	2.31		
		Postgraduate (d)	294	2.19	1.10	2.20	2.50		
**Employment Status**	Conspiracy Theories	Full-time (a)	737	2.84	1.00	2.77	2.92	0.001*	e > a
		Part-time (b)	54	2.86	1.05	2.99	3.57		
		Retired (c)	53	2.89	0.91	2.64	3.14		
		Student (d)	230	3.01	1.01	2.87	3.14		
		Unemployed (e)	142	3.17	1.00	3.00	3.34		
	Environmental Factors	Full-time (a)	737	2.81	0.88	2.75	2.88	0.740	
		Part-time (b)	54	2.84	0.78	2.63	3.06		
		Retired (c)	53	2.89	0.84	2.72	3.18		
		Student (d)	230	2.88	0.79	3.00	3.21		
		Unemployed (e)	142	2.80	0.85	2.66	2.95		
	Faith Factors	Full-time (a)	737	2.23	1.05	2.15	2.30	0.000*	e > a
		Part-time (b)	54	2.68	1.17	2.36	3.00		
		Retired (c)	53	2.82	1.34	2.46	3.19		
		Student (d)	230	2.49	1.04	2.35	2.62		
		Unemployed (e)	142	2.97	1.15	2.78	3.17		

* *p* < 0.05 n, M, SD, LL, and UL represent Number, Mean, Standard Deviation, Lower Limit, and Upper Limit, respectively

## Discussion

The present study aimed at investigating the relationship between perception of causes of COVID-19, attitudes towards vaccine and trust in information sources from an infodemic perspective, was conducted with 1216 participants from seven geographical regions of Turkey.

The study results demonstrated that while slightly more than half of the participants displayed a positive attitude towards getting the COVID-19 vaccine, about a third had vaccine hesitancy. According to the May 2021 report of the Johns Hopkins Coronavirus Resource Center, considering that Turkey ranks fifth in terms of the number of confirmed cases in the world, this high rate of undecided people makes the situation riskier [2]. In other words, this desperate picture of COVID-19 shows that vaccine hesitancy in the Turkish sample is worrying. The most important information supporting this view is the fact that 55 to 85% of the society should be vaccinated to prevent the spread of Covid-19 infection [23, 24]. In a study in which Turkey and the United Kingdom were compared, approximately one out of three people in Turkey, which is consistent with our results, and one out of seven people in the United Kingdom were undecided about getting the COVID-19 vaccine [25]. In a study conducted in France, approximately 29% of the participants did not want to have the COVID-19 vaccine [26]. In a systematic review, it is reported that vaccine hesitancy is increasing worldwide [27]. In general, it is known that perceived risks and benefits, certain religious beliefs, lack of knowledge and awareness levels have led to this increase [28].

It is also known that societies’ attitudes towards vaccination vary from one country to another. Thus, to identify the leading causes of anti-vaccination attitudes in societies and countries, a global survey should be conducted [29]. Especially in countries where the COVID-19 vaccine is being introduced, care should be taken to increase confidence in COVID-19 vaccines and to minimize infodemic [30]. Of course, at this point, it is also critical to know which information sources are more reliable for individuals on accessing information about COVID-19. For example, in a study conducted during the Ebola epidemic, tweets containing misleading medical information reached approximately 15 million potential readers in just 1 week [15]. Therefore, it is obvious that spread of infodemic by these and similar means will have negative effects on vaccination decisions. It is also known that exposure to anti-vaccine blogs and websites negatively affects vaccination intention [31].

In the present study, while the participants who stated that they would be vaccinated mostly trusted YouTube, those who stated that they would not be vaccinated trusted WhatsApp groups most and those who were undecided trusted social media platforms such as Facebook, Twitter, and Instagram. However, the results of the Chi-Square analysis demonstrated that YouTube or WhatsApp groups did not have a significant effect on the participants’ attitudes towards vaccine. On the other hand, it was concluded that the participants who stated that they did not trust social media platforms such as Facebook, Twitter and Instagram displayed a positive attitude towards vaccine. Perhaps the most noteworthy result of the study was that of the information sources, government institutions and health professionals had the most significant impact on individuals’ attitudes of vaccination. Trusting government institutions, and health authorities and experts is critical in reducing vaccine resistance [32].

The role of infodemic in the development of vaccine resistance cannot be ignored. Infodemic, which came onto the agenda again with the COVID-19 pandemic, a great amount information some of which is true, some of which is false emerges during an epidemic, spreads rapidly like a virus and complicates the organization of health [6]. As is reported in previous studies, there are hesitations about the origin of the virus, and false information is presented as correct on various platforms, which supports conspiracy theories and ultimately affects the decision to get vaccine [3, 33–35]. The participants in the present study mostly perceived conspiracy theories as the causes of Covid-19, followed by the perception of environmental factors and faith factors. Therefore, it is possible to state that conspiracy theories constitute the greatest part of individuals’ perception of the causes of COVID-19. Similarly, in another study, 18% of the participants in Turkey and 12% of those in the United Kingdom thought the virus was of artificial origin [25]. On the other hand, according to the results of a study conducted in Nigeria, these theories are considered as the exaggeration of governments / media, China’s biological weapon, the strategy to control the population, and the plague of the modern age due to sins committed by people [12]. Based on this, it can be stated that as the perspectives of individuals on the causes of COVID-19 change, so do their attitudes towards vaccination. For instance, in the present study, individuals who perceived conspiracy theories and belief factors as the causes of COVID-19 had the intention to be vaccinated. Although this result is interesting, a social perception that the virus comes from a human or a divine source may have increased individuals’ desire to be vaccinated. On the other hand, as their age progressed, people thought that this virus was caused by an ecological / environmental problem and the attitude towards vaccination became negative, which may be related to the increase in the awareness levels of the individuals and the decrease in their life satisfaction as they age.

Of the participants, for those who lived in Van, a city in the east of Turkey, those who were primary school graduates and those who were unemployed, the coronavirus is originated from conspiracy theories compared to those who lived in Izmir, a city in the west of Turkey, those who had the master’s degree and those who worked full-time respectively. It will be useful to conduct studies that are more detailed in the future and to examine the other possible reasons underlying this result. In the present study, according to the female participants, single participants and the participants not having a diagnosis of COVID-19 positive, the coronavirus was originated from environmental factors, which might be due to the fact that ecological awareness of these participants was higher than was that the other participants. Of the participants, for those who were diagnosed with COVID-19 or had a relative diagnosed with COVID-19, those who lived in Şanlıurfa, a city in southeastern Turkey, those who were primary school graduates and those who were unemployed, the coronavirus is originated from faith factors compared to those who were not diagnosed with COVID-19 or did not have a relative diagnosed with COVID-19, those who lived in Izmir, a city in the west of Turkey, those who had the master’s degree and those who worked full-time respectively. This result can be said to reflect an understanding originating from prehistoric times, because, in those ages, it was believed that the causes of diseases were mostly based on a divine source and that individuals who got sick were punished by God. For example, it is stated that in ancient civilizations such as Sumer, Babylon, Assyria and Hittites, it was believed that there was a relationship between sin and disease, and that those who did not obey religious orders would suffer the wrath of God [36].

In some studies in the literature, perceptions about the causes of COVID-19 are investigated in terms of the level of knowledge. According to the results of a study conducted in Malaysia within this context, of the participants, those who were of Chinese ethnicity, were middle-aged, were university graduates, had health-related education, and had high income levels had a better knowledge of COVID-19 [37]. At this stage, the present study is of importance because it sheds light on the current literature although it lacks empirical evidence focusing on statistical differences between the perception of the causes of COVID-19 and the descriptive characteristics of individuals, which makes the interpretation of the results difficult. On the other hand, the assumption that the participants selected from seven different geographical regions represent Turkey, that the answers given to the questionnaires reflect their real attitude and that all the participants knew how to respond to e-questionnaires constitute the main limitations of the present study. It may be beneficial to plan similar studies with larger sample sizes in order to compare the findings obtained in this study with those of the studies from different cultures.

## Conclusion

In the present study, carried out to investigate the relationships between the participants’ perceived causes of COVID-19, their attitudes towards vaccine and their levels of trust in information sources from the perspective of infodemic, it was concluded that vaccine hesitancy in Turkey was at an alarming rate. It was observed that the source of information trusted most was YouTube for the participants who stated that they would be vaccinated, WhatsApp groups for the participants who stated that they would not be vaccinated and social media for the participants who stated that they were undecided. On the other hand, it was determined that there was no significant relationship between the level of trust in YouTube and WhatsApp and the participants’ attitudes towards vaccine. Of the participants, those whose level of trust in government institutions and health professionals was high displayed significantly more favorable attitudes towards vaccine. The participants obtained the highest mean score from the Conspiracy Theories (attempt to sell drugs and vaccines, biological weapons, a big experiment …) subscale of the PCa-COVID-19 scale. It was concluded that those who lived in Van, a city in the east of Turkey, those who were primary school graduates and those who were unemployed believed in conspiracy theories more. It was determined that the higher the participants’ perception of conspiracy theories and faith factors was, the more willing they were to be vaccinated. This suggests that the perception that the virus comes from human and divine sources may had increased the participants’ desire to be vaccinated. This result may seem quite surprising because, in an environment where it is believed that the virus is not of natural origin, individuals’ displaying a negative attitude towards the vaccine is something expected. On the other hand, that a social perception that the virus emerged from conspiracy and belief factors brings about a risk which will turn the social perception into an infodemic difficult to fight in the future. Therefore, all health institutions, especially health professionals, assume serious responsibilities in guiding the target audience immediately and accurately through scientific means. In addition, initiating a global health literacy campaign in order for people to know which sources of information on COVID-19 and vaccines are trustable could provide useful gains.

## Supplementary Information

**Additional file 1.**

## Data Availability

The datasets used and/or analysed during the current study are available from the corresponding author on reasonable request.

## References

[1] Mheidly N, Fares J (2020). Leveraging media and health communication strategies to overcome the COVID-19 infodemic. J Public Health Policy.

[2] Johns Hopkins Coronavirus Resource Center **,** https://coronavirus.jhu.edu/map.html

[3] Loomba S, Figueiredo A, Piatek SJ, Graaf K, Larson HJ (2021). Measuring the impact of COVID-19 vaccine misinformation on vaccination intent in the UK and USA. Nat Hum Behav.

[4] Gölbaşı S, Metintas S (2020). COVID-19 pandemic and infodemic. ESTUDAM Public Health J.

[5] Munich Security Conference: WHO; 15 February 2020 https://www.who.int/dg/speeches/detail/munich-security-conference. Accessed 15 Feb 2021.

[6] 1st WHO Infodemiology Conference (2020). https://www.who.int/news-room/events/detail/2020/06/29/default-calendar/pre-conference-1st-who-infodemiology-conference Accessed 15 Feb 2021.

[7] Fernández-Torres MJ, Almansa-Martínez A, Chamizo-Sánchez R (2021). Infodemic and Fake News in Spain during the COVID-19 Pandemic. Int J Environ Res Public Health.

[8] Chong YY, Cheng HY, Chan HYL, Chien WT, Wong SYS (2020). COVID-19 pandemic, infodemic and the role of eHealth literacy. Int J Nurs Stud.

[9] Zhang X, Zhang ZK, Wang W, Hou D, Xu J, Ye X, et al. Multiplex network reconstruction for the coupled spatial diffusion of infodemic and pandemic of COVID-19. Int J Digital Earth. 2021;14(4):401–23. 10.1080/17538947.2021.1888326.

[10] Tanne JH, Hayasaki E, Zastrow M, Pulla P, Smith P, Rada AG. Covid-19: how doctors and healthcare systems are tackling coronavirus worldwide. BMJ. 2020:m1090. 10.1136/bmj.m1090. 10.1136/bmj.m1090 32188598

[11] Uscinski JE, Enders AM, Klofstad C, Seelig M, Funchion J, Everett C, et al. Why do people believe COVID-19 conspiracy theories? Harvard Kennedy School Misinformation Rev. 2020;1(3). 10.37016/mr-2020-015.

[12] Olatunji OS, Ayandele O, Ashirudeen D, Olaniru OS (2020). “Infodemic” in a pandemic: COVID-19 conspiracy theories in an african country. Social Health Behav.

[13] Mourad A, Srour A, Harmanai H, Jenainati C, Arafeh M (2020). Critical impact of social networks infodemic on defeating coronavirus COVID-19 pandemic: twitter-based study and research directions. IEEE Trans Netw Serv Manag.

[14] Hoşgör H, Aközlü Z (2021). Investigation of knowledge, compliance with prevention and risk perception levels about covid-19 in terms of sociodemographic characteristics of students in health programs. Int J Health Manage Strategies Res.

[15] Oyeyemi SO, Gabarron E, Wynn R (2014). Ebola, twitter, and misinformation: a dangerous combination?. BMJ.

[16] World Health Organization (WHO). Coronavirus disease (COVID-19) outbreak situation, 2020. https://www.who.int/emergencies/diseases/novel-coronavirus-2019 Accessed 15 Feb 2021.

[17] Alpyıldız G, Aslan D. Strugglemethods on mis information about Novel Coronavirus Disease (COVID-19). Mersin Üniv Saglık Bilim Derg. 2020. 10.26559/mersinsbd.763836.

[18] Hoşgör H, Aközlü ZG, Hoşgör D (2021). The perception concerning the COVID-19 pandemic: case of Turkey. Eur Res J.

[19] Gürbüz S, Şahin F (2016). Sosyal Bilimlerde Araştırma Yöntemleri.

[20] Geniş B, Gürhan N, Koç M, Geniş Ç, Şirin B, Çırakoğlu OC, et al. Development of perceptıon and attitude scales related with Covıd-19 pandemıa. Pearson J Soc Sci Human. 2020. 10.46872/pj.127.

[21] Çırakoğlu OC (2011). Investigation of perceptions associated with swine flu (H1N1) epidemic in the context of variables of anxiety and avoidance. Turk Psikoloji Dergisi.

[22] Tabachnick BG, Fidell LS. Experimental designs using ANOVA: Thomson/Brooks/Cole; 2007.

[23] Sanche S, Lin YT, Xu C, Severson ER, Hengartner N, Ke R (2020). High contagiousness and rapid spread of severe acute respiratory syndrome coronavirus 2. Emerg Infect Dis.

[24] Kwok KO, Lai F, Wei WI, Wong SYS, Tang JWT (2020). Herd İmmunity—estimating the level required to halt the Covıd-19 epidemics İn affected countries. J Inf Secur.

[25] Salali GD, Uysal MS. COVID-19 vaccine hesitancy is associated with beliefs on the origin of the novel coronavirus in the UK and Turkey. Psychol Med. 2020:1–3. 10.1017/S0033291720004067. 10.1017/S0033291720004067 PMC7609204 33070804

[26] Schwarzinger M, Watson V, Arwidson P, Alla F, Luchini S (2021). COVID-19 vaccine hesitancy in a representative working-age population in France: a survey experiment based on vaccine characteristics. Lancet Public Health.

[27] Lin C, Tu P, Beitsch LM (2020). Confidence and receptivity for COVID-19 vaccines: a rapid systematic review. Vaccines.

[28] Sallam M (2021). COVID-19 vaccine hesitancy worldwide: a concise systematic review of vaccine acceptance rates. Vaccines..

[29] Erkekoğlu P, Köse SBE, Balcı A, Yirün A (2020). Vaccine hesitancy and effects of COVID-19. J Lit Pharm Sci.

[30] Malik AA, McFaddena SM, Elharake J, Omer SB (2020). Determinants of Covıd-19 vaccine acceptance İn the us. EClinicalMedicine..

[31] Nan X, Madden K (2012). (2012). HPV vaccine information in the blogosphere: how positive and negative blogs influence vaccine-related risk perceptions, attitudes, and behavioral intentions. Health Commun.

[32] Murphy J, Vallières F, Bentall RP, Shevlin M, McBride O, Hartman TK, et al. Psychological characteristics associated with COVID-19 vaccine hesitancy and resistance in Ireland and the United Kingdom. Nat Commun. 2021;12(1):29. 10.1038/s41467-020-20226-9. 10.1038/s41467-020-20226-9 PMC7782692 33397962

[33] Akyüz SS (2020). Misinformation outbreak: fake news circulation in Turkey during COVID-19 pandemic. Akdeniz Üniversitesi İletişim Fakültesi Dergisi.

[34] Sallam M, Dababseh D, Eid H, Al-Mahzoum K, Al-Haidar A, Taim D, et al. HighRates of COVID-19 vaccine Hesitancyand its association with ConspiracyBeliefs: a study in Jordan andKuwait among other Arab countries. Vaccines. 2021;9(1). 10.3390/vaccines9010042. 10.3390/vaccines9010042 PMC7826844 33445581

[35] Brennen JS, Simon F, Howard PN, Nielsen RN (2020). Types, Sources, and Claims of COVID-19 Misinformation. Reuters Institute Report, Factsheet.

[36] Atmaca V (2010). The relationship of illness and sin in old civilizations and the matter of God’s revenge. Atatürk Üniversitesi İlahiyat Fakültesi Dergisi.

[37] Chang CT, Lee M, Lee JCY, Lee NCT, Ng TY, Shafie AA (2021). Public KAP towards COVID-19 and antibiotics resistance: a malaysian survey of knowledge and awareness. Int J Environ Res Public Health.

