# Supplementary material for: Investigation of the relationships between perceived causes of COVID-19, attitudes towards vaccine and level of trust in information sources from the perspective of Infodemic: the case of Turkey
Source: BMC Public Health. 2021 Jun 23;21:1195. doi: 10.1186/s12889-021-11262-1 (PMC8219470; doi:10.1186/s12889-021-11262-1)
Supplement: Supplementary file 1 — Additional file 1. [file 12889_2021_11262_MOESM1_ESM.docx]

**Appendix**

**Investigation of the Relationships between Perceived Causes of COVID-19, Attitudes towards Vaccine and Level of Trust in Information Sources from the Perspective of Infodemic: The Case of Turkey**

**Descriptive Characteristics of the Participants**

1. **Age:**  a. 18-25 b. 26-33 c. 34-41 d. 42-49 e. ≥ 50
2. **Gender:** a. Female b. Male
3. **Marital status:** a. Married b. Single
4. **City of residence:** a. Istanbul b. Ankara c. Izmir d. Adana e. Samsun f. Şanlıurfa g. Van
5. **Educational status:** a. Primary education b. High School c. Bachelor’s degree d. Postgraduate
6. **Employment status:** a. Full-time b. Part-time c. Retired d. Student e. Unemployed
7. **Have you ever been diagnosed with Covid-19?**: a. Yes b. No
8. **Has any of your relatives been diagnosed with Covid-19?:** a. Yes b. No
9. **Has any of your relatives died due to Covid-19?:** a. Yes b. No

**Covid-19 Vaccine Attitude**

1. **Are you considering getting the Covid-19 Vaccine?:** a. Yes b. No c. Undecided

**Cross Tabulations**

**1.** Please mark your TRUST level in the resources specified below in order to get INFORMATION ABOUT COVID-19.

|  | **Information Sources** | **NEVER trust (1)** | **MODERATELY trust (2)** | **HIGHLY Trust (3)** |
| --- | --- | --- | --- | --- |
| ***1*** | Social media |  |  |  |
| ***2*** | YouTube |  |  |  |
| ***3*** | WhatsApp Groups |  |  |  |
| ***4*** | Websites |  |  |  |
| ***5*** | Newspaper |  |  |  |
| ***6*** | Television |  |  |  |
| ***7*** | Friends and Relatives |  |  |  |
| ***8*** | Government Agencies |  |  |  |
| ***9*** | Healthcare Professionals |  |  |  |

*** Although the data of the study were collected in this way, only the most trust information sources and vaccination attitude were considered within the scope of the article.*

**Perception of Causes of COVID-19 (PCa-COVID-19) Scale:**

Read each item carefully and mark the opinion that is most applicable to you.

| **Items** | | **Strongly**  **Disagree (1)** | **Disagree (2)** | **Undecided (3)** | **Agree (4)** | **Strongly**  **Agree (5)** |
| --- | --- | --- | --- | --- | --- | --- |
| ***1*** | This disease is a political game that is run by developed countries. |  |  |  |  |  |
| ***2*** | The cause of this pandemic is the effort of developed countries to sell drugs and vaccines. |  |  |  |  |  |
| ***3*** | This virus is spread deliberately in order to contribute to the economic system. |  |  |  |  |  |
| ***4*** | This disease was developed as a biological weapon. |  |  |  |  |  |
| ***5*** | This pandemic is part of a large experiment. |  |  |  |  |  |
| ***6*** | The reason for this disease is economic crisis. |  |  |  |  |  |
| ***7*** | Environmental pollution is one of the major causes of the disease. |  |  |  |  |  |
| ***8*** | One of the causes of the pandemic is the contamination of water resources. |  |  |  |  |  |
| ***9*** | This disease is the result of an unhealthy lifestyle. |  |  |  |  |  |
| ***10*** | Global warming is one of the causes of the pandemic. |  |  |  |  |  |
| ***11*** | These pandemics are an effort to balance nature. |  |  |  |  |  |
| ***12*** | These types of pandemics are punishments from God for the backsliding of society away from religion. |  |  |  |  |  |
| ***13*** | This pandemic is the indignation of God against social degradation. |  |  |  |  |  |
| ***14*** | This pandemic is our destiny. |  |  |  |  |  |
